# Revealing heterogeneous nucleation of primary Si and eutectic Si by AlP in hypereutectic Al-Si alloys

**DOI:** 10.1038/srep25244

**Published:** 2016-04-28

**Authors:** Jiehua Li, Fredrik S. Hage, Xiangfa Liu, Quentin Ramasse, Peter Schumacher

**Affiliations:** 1 Institute of Casting Research, Montanuniversität Leoben, Leoben, A-8700, Austria; 2 SuperSTEM Laboratory, SciTech Daresbury Campus, Keckwick Lane, Daresbury, WA4 4AD, UK; 3 Key Laboratory for Liquid-Solid Structural Evolution and Processing of Materials, School of Materials Science and Engineering, Shandong University, Jinan 250061, China; 4 Austrian Foundry Research Institute, Leoben, A-8700, Austria

## Abstract

The heterogeneous nucleation of primary Si and eutectic Si can be attributed to the presence of AlP. Although P, in the form of AlP particles, is usually observed in the centre of primary Si, there is still a lack of detailed investigations on the distribution of P within primary Si and eutectic Si in hypereutectic Al-Si alloys at the atomic scale. Here, we report an atomic-scale experimental investigation on the distribution of P in hypereutectic Al-Si alloys. P, in the form of AlP particles, was observed in the centre of primary Si. However, no significant amount of P was detected within primary Si, eutectic Si and the Al matrix. Instead, P was observed at the interface between the Al matrix and eutectic Si, strongly indicating that P, in the form of AlP particles (or AlP ‘patch’ dependent on the P concentration), may have nucleated on the surface of the Al matrix and thereby enhanced the heterogeneous nucleation of eutectic Si. The present investigation reveals some novel insights into heterogeneous nucleation of primary Si and eutectic Si by AlP in hypereutectic Al-Si alloys and can be used to further develop heterogeneous nucleation mechanisms based on adsorption.

Al–Si based alloys are the most widely used Al based foundry alloys due to their excellent combination of mechanical properties and castability. For hypoeutectic Al-Si based alloys, the modification of eutectic Si can have a significant impact on the mechanical properties, in particular the dynamic fatigue properties and has therefore been widely investigated since 1921^1^. In terms of the growth of eutectic Si, it is generally accepted that the so-called impurity-induced twinning (IIT) mechanism^2^ and the twin plane re-entrant edge (TPRE) mechanism^3^,^4^ as well as poisoning of the TPRE mechanism^5^ are active under certain conditions and contribute to the modification process. Recently, the concept of solute entrainment was also introduced to interpret the observations of eutectic modification^6^,^7^,^8^,^9^. However, in terms of the nucleation of eutectic Si, there is still a lack of detailed research to elucidate the interaction between modifying agents (e.g. Sr, Na) with nucleation sites (e.g. AlP)^10^,^11^,^12^,^13^,^14^,^15^,^16^,^17^,^18^,^19^. AlP is thought to be a possible heterogeneous nucleation site for eutectic Si due to its excellent lattice match with Si^11^,^12^,^13^. Nogita *et al.*^11^ found evidence of centrally located AlP particles surrounded by a Si crystal in a hypoeutectic Al-Si alloy containing 40 ppm P. Similar results were also obtained by Ho and Cantor^14^ in entrained droplet experiments^15^. It has been reported that as little as 0.25–2 ppm P is sufficient to form AlP which could act as a heterogeneous nucleation site for eutectic Si^14^, verifying the results by Crosley and Mondolfo^10^ and Flood and Hunt^16^. As a modifying agent, Na has been reported to cause a poisoning effect in P containing hypoeutectic Al-Si alloys^10^. The addition of Na forces the nucleation of Si to larger undercoolings (lower nucleation temperatures), which was attributed to the formation of Na_3_P compounds and thereby the presence of a smaller amount of the active AlP phase. Furthermore, similar to the poisoning effect of Na, Sr was also reported to have a poisoning effect on the AlP compound^6^,^17^. It was proposed that intermetallic compounds such as Al_2_Si_2_Sr^17^ or Sr_3_P_2_^6^ consumed the AlP, thus reducing the number of nucleated Si particles. A combined thermodynamic computation and phase-field simulation was also used to investigate the impact of P and Sr on solidification sequence and morphology of hypoeutectic Al-Si alloys^18^. Clearly, there is an important interaction between the modifying agents (e.g. Sr, Na) and the nucleation sites (e.g. AlP). Elucidating the heterogeneous nucleation of eutectic Si by AlP is, therefore, of great importance, in order to avoid or reduce the poisoning effects of modifying agents (if any) and optimize the solidification microstructure of hypoeutectic Al-Si alloys.

Similarly, for hypereutectic Al-Si alloys, apart from the modification of eutectic Si, the refinement of primary Si is also believed to be a key factor in improving properties and castability^19^,^20^. To date, as for the hypoeutectic case, it is generally accepted that AlP is a perfect site for the heterogeneous nucleation of primary Si due to its excellent lattice match with Si^11^,^12^,^13^. Here, the refinement of primary Si is generally achieved by the addition of different P-rich master alloys, e.g. Al-Si-P^21^,^22^. The refinement potency and P recovery is mainly related to the morphology, size and quantity of AlP particles in the master alloy, which could influence the dissolution rate of AlP and thereby affect the refining performance^22^. It has been reported that the refinement potency and P recovery of the Al-15Si-3.5P master alloy is much higher than that of a Cu-8.5P master alloy due to the pre-formed AlP particles. Clearly, the formation of AlP particles is of great importance for enhancing the refinement potency. However, the formation mechanisms of such AlP particles are still under debate. Depending on the P concentration and solidification conditions, P can be present in different forms (e.g. AlP cluser, AlP patch and/AlP particles)^6^. The distribution of P, in particular at the atomic scale, still remains to be explored, due in particular to difficulties in detecting P accurately using transmission electron microscopy (TEM) based techniques necessary for atomic scale studies. The reasons for these difficulties can be threefold: (i) all atomic numbers are very close (Al: 13, Si: 14, P: 15), which makes it challenging to use atomic number (Z) contrast for composition analysis in high resolution high angle annular dark field (HAADF) scanning transmission electron microscopy (STEM) imaging, (ii) P is very likely to react with O_2_ (oxidation) and/or water during sample preparation, and (iii) the P concentration is usually at a very trace level (e.g. less than 10 ppm), close to or beyond the detection limits of these characterisation techniques.

In this paper, HAADF STEM imaging and electron energy loss spectroscopy (EELS) were used to elucidate the distribution of P in hypereutectic Al-Si-P alloys. The aim of this investigation is to elucidate the heterogeneous nucleation of primary Si and eutectic Si by AlP in hypereutectic Al-Si alloys and to further develop the heterogeneous nucleation mechanisms based on adsorption. It should be noted here that the investigation in hypereutectic Al-Si based alloys can also be used to interpret the heterogeneous nucleation of eutectic Si in hypoeutectic Al-Si based alloys, although the P concentration in hypoeutectic Al-Si based alloys is usually much lower than that in hypereutectic Al-Si based alloys, guiding the choice of the hypereutectic case for this study.

## Results

**Figure 1:**
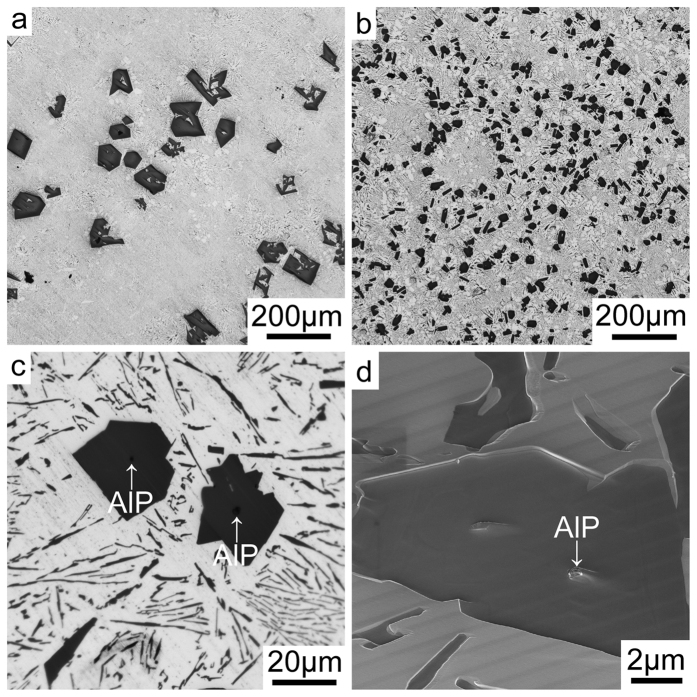
Microstructure of Al-18Si without and with 0.03 P. (**a**) Optical microscopy image of Al-18Si alloy, (**b**). Optical microscopy image of Al-18Si-0.03P alloy, (**c**) Optical microscopy image of Al-18Si-0.03P alloy, enlarged from (**b**) to show the AlP particle in the centre of primary Si, as marked with a white arrow, (**d**) SEM image of Al-18Si-0.03P alloy showing the AlP particle in the centre of primary Si, as marked with a white arrow. This AlP particle was lifted out using FIB for further TEM sample preparation.

**Figure 2:**
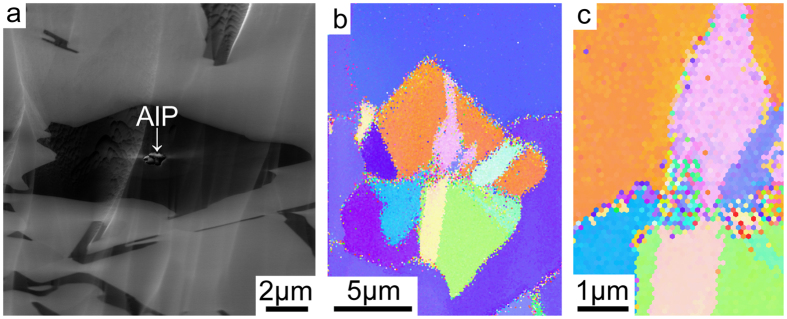
(**a**) SEM image of Al-18Si-0.03P alloy, (**b**) EBSD map of primary Si in Al-18Si-0.03P alloy, (**c**) is enlarged from (**b**). The primary Si has multi-fold branched orientation, even within one primary Si.

Figure 1 shows the as-cast microstructure of Al-18Si without and with the addition of 0.03P. The presence of 0.03P results in a significant increase in the number density of primary Si and a significant decrease of the size of the primary Si, as clearly seen when comparing Fig. 1a,b. AlP particles were very often observed in the centre of primary Si: typical examples are marked with a white arrow in Fig. 1c. Conventional focused ion beam (FIB) ‘lift out’ techniques were used to attempt the extraction of one such AlP particle within the primary Si region imaged in Fig. 1d, marked with a white arrow, for further TEM sample preparation and characterisation. However, during lift-out and subsequent thinning, the AlP particle itself was removed, leaving only the surrounding primary Si. No further AlP particle was observed within the primary Si after final thinning, indicating that AlP particles located in the centre of primary Si are relatively small in size. The small size of such AlP particles makes it very challenging to determine their orientation relationship with the primary Si using electron back scattered diffraction (EBSD), as shown in Fig. 2. However, EBSD could be used to determine the orientation of grains within single primary Si areas. The primary Si imaged in Fig. 2 has multi-fold branched orientation, which is fully consistent with previous observations of Si nucleated on the partly solidified Si substrate^23^,^24^.

**Figure 3:**
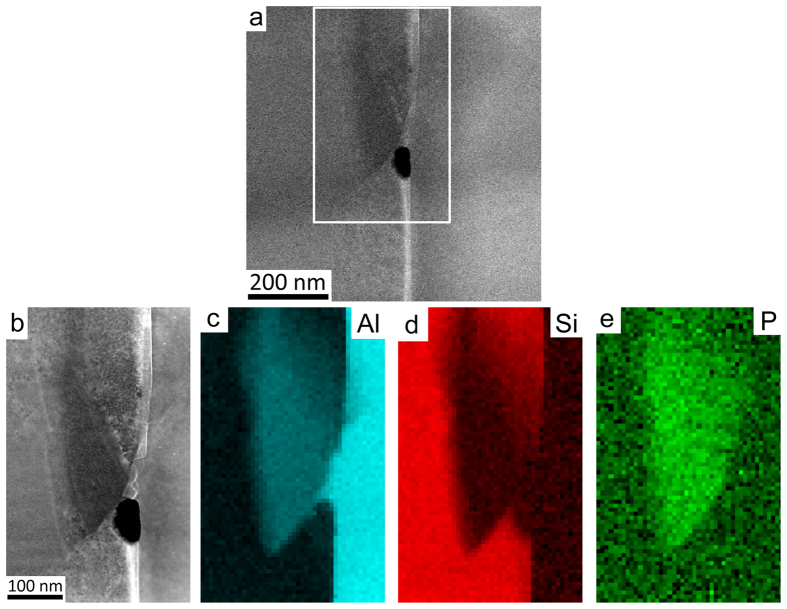
High resolution HAADF STEM images (a,b) and EELS maps of Al (c), Si (d) and P (e) in Al-18Si-0.03P alloy. The AlP particle was observed at the interface between the Al matrix and eutectic Si.

Despite the absence of AlP particles within primary Si in the final FIB sample, the sample shows a number of interesting structural features. In particular, small AlP particles were observed at the interface between eutectic Si and the Al matrix. Figure 3 shows high resolution HAADF STEM images (Fig. 3a,b) and EELS maps of Al (Fig. 3c), Si (Fig. 3d) and P (Fig. 3e) of one such AlP particle in Al-18Si-0.03P alloy. As the HAADF-STEM contrast scales to a good approximation with the atomic number Z of the observed material as ~Z^1.7^, no significant contrast was observed in the HAADF STEM images (Fig. 3a,b) due to the very close atomic number of Al (13), Si (14) and P (15). However, the presence of P can be clearly confirmed by the chemical maps obtained with EELS (Fig. 3e) (see methods section). The geometry of the P-rich region revealed by these EELS maps indicates that the AlP particle is likely to have initially nucleated on the surface of the Al matrix.

**Figure 4:**
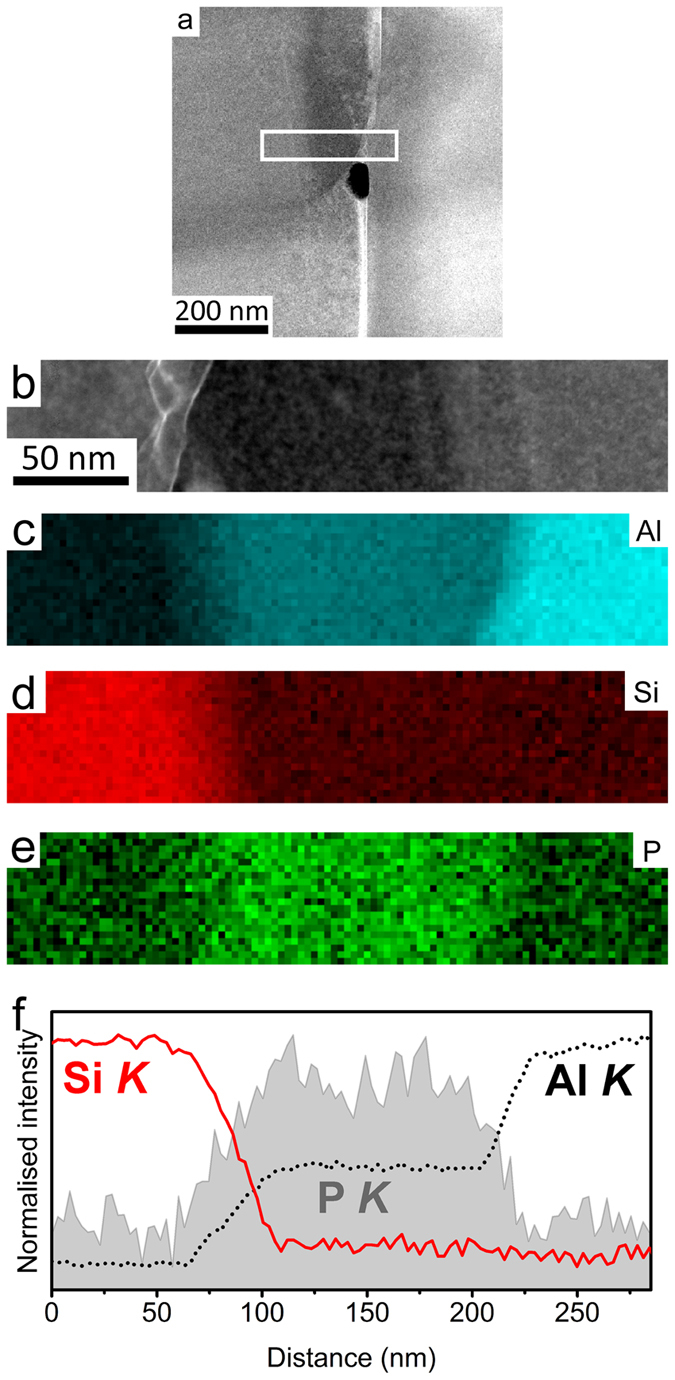
High resolution HAADF STEM images (a,b), EELS maps of Al (c), Si (d), P (e) and line scanning analysis of Al, Si, P (f) in Al-18Si-0.03P alloy. The AlP particle was again observed at the interface between the Al matrix and eutectic Si.

The presence of P at the interface between the Al matrix and eutectic Si can be more clearly seen in Fig. 4f where the EELS signal intensity is averaged across the interface and displayed as a line profile. It should be noted that Fig. 4a shows the same area as that mapped in Fig. 3, but the EELS dataset from which the maps in Fig. 4b,c, were acquired at a higher magnification in order to show more details at the interface between Al matrix and eutectic Si. From Fig. 4f, a simultaneous increase of Al and P strongly indicates that the AlP particle forms at the interface between Al matrix and eutectic Si. As proposed in^6^, an AlP patch and/or AlP particle is more likely to be adsorbed on the surface of the Al matrix and then acts as a heterogeneous nucleation site for eutectic Si. Due to the very limited solubility of P within Al (e.g. 40 ppm)^25^,^26^ and Si, no significant amount of P was detected within eutectic Si and the Al matrix, as clearly shown in Figs 3 and 4.

**Figure 5:**
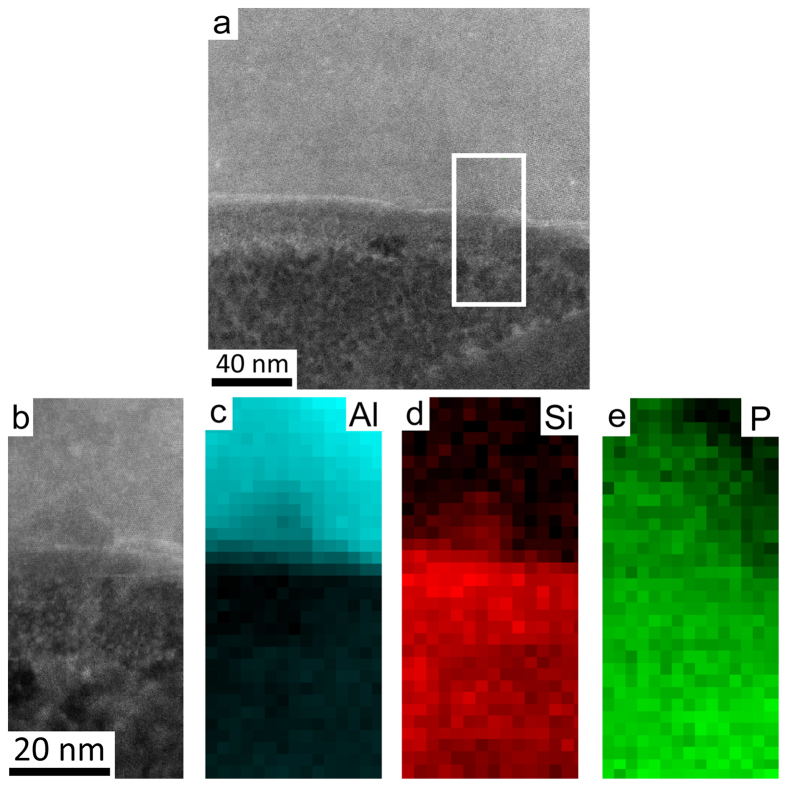
High resolution HAADF STEM images (a,b) and EELS maps of Al (c), Si (d) and P (e) in Al-18Si-0.03P alloy. The Al matrix was tilted to <011>_Al_ zone axis. A significant amount of P was observed to be distributed uniformly at the interface between the Al matrix and eutectic Si, which is due to an overlap between the analysed area and the AlP particle.

**Figure 6:**
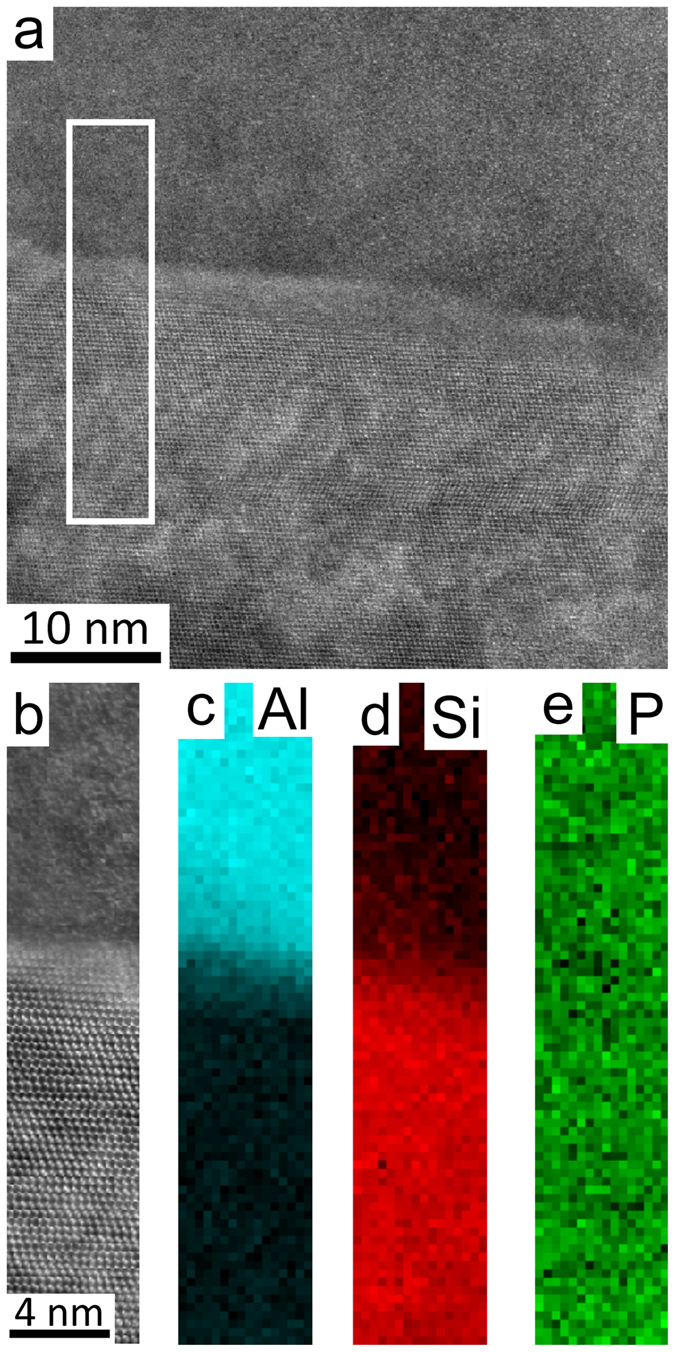
High resolution HAADF STEM images (a,b) and EELS maps of Al (c), Si (d) and P (e) in Al-18Si-0.03P alloy. The Si particle was tilted to <011>_Si_ zone axis. A significant amount of P was observed to be distributed uniformly at the interface between the Al matrix and eutectic Si, which is due to an overlap between the analysed area and the AlP particle.

In order to further elucidate the distribution of P, the Al matrix was tilted to the <011>_Al_ zone axis and the exact same area was observed again, as shown in Fig. 5. A significant amount of P was observed to be distributed uniformly at the interface between the Al matrix and eutectic Si, due to an overlap between the analysed area and the AlP particle. However, high resolution imaging revealed no special orientation relationship between the AlP and the Al matrix: with the Al matrix on axis, the AlP particle was far away from any zone axis, which can be further supported by the high resolution STEM image (see Supplementary Figure S1). Similarly, the eutectic Si particle was also tilted to <011>_Si_ zone axis, as shown in Fig. 6. Again, a significant amount of P was observed to be distributed uniformly at the interface between the Al matrix and eutectic Si. This time however, a typical cubic to cubic orientation relationship was observed between the AlP and eutectic Si, as shown in Fig. 6a,b, strongly indicating that AlP is an efficient heterogeneous nucleation site for eutectic Si. However, no significant amount of P was observed within the eutectic Si particle (see Supplementary Figure S2). This strongly indicates that, in as-cast conditions, P is more likely to be in the form of AlP particles, which is fully consistent with the solubility of P in Al being very low (e.g. 40 ppm)^25^,^26^ and the presence of Si up to 18 wt.% as well as other possible impurities (e.g. Fe, Cu) do not affect significantly the P solubility^27^. This can be further supported by melt spun experimental results (as shown in Supplementary Figure S3).

The cooling rate during melt spinning is mainly dependent on the heat transfer coefficient, process parameters and contact time/length between metallic melt or metallic ribbon and substrate, respectively. The heat transfer coefficient between the melt and the chill wheel and thermal diffusivity of the wheel material has a more significant influence on cooling rate of the ribbon. For the cooling rate estimation of melt spinning, a calculation (10 ^6^ K/s) has been developed and widely used^28^. Furthermore, it should be noted that the solubility of P is mainly depended on the meting temperature. For example, at higher melting temperature (i.e. 850 °C in the present investigation), more P (i.e. more than 300 ppm) can be dissolved into the melting, which is strongly supported by the Figure S3. No significant presence of AlP particles was observed in melt-spun condition. With decreasing the melting temperature (i.e. 720 °C for conventional casting temperature), the solubility of P is also greatly decreased (i.e. 10 ppm). In the case of higher melting temperature, the increase of cooling rate (i.e. 10^6^ °C/s during melting spinning) can “freeze” the “dissolved P” into the α-Al matrix. However, it should be noted that, in the case of lower melting temperature, the increase of cooling rate does not cause any significant effect on the solubility of P because of the fact that P cannot be “dissolved” into the α-Al matrix. Furthermore, the presence of P appears to affect the heterogeneous nucleation of primary Si and/or eutectic Si, but does not affect the growth of primary Si and/or eutectic Si. As shown in Figure S4 (Supplementary Information), significant Si twining was also observed, indicating that the TPRE mechanism is active under these conditions.

## Discussion

The existence of P in hypereutectic Al-Si alloys can result in three scenarios: (i) P is randomly distributed within the Al matrix. Considering the very low solubility of P in Al, the random P distribution may only be possible when the P concentration is at a trace level (e.g. 0.44 ppm in high purity Al-Si alloy^6^). (ii) P combines with Al in the form of small AlP clusters or AlP patches within the Al matrix when the P concentration is in the range of 0.44 ppm to 3 ppm^6^. When AlP clusters or AlP patches reach a critical size at defined undercoolings, the heterogeneous nucleation of primary Si and/or eutectic Si is possible. (iii) P combines with Al in the form of large AlP particles within the Al matrix when the P concentration is more than 3 ppm^6^. In the present investigation, the P concentration is measured at 0.03 wt% (300 ppm), indicating that P is more likely to combine with Al in the form of large AlP particles within the Al matrix. Indeed, the size of AlP particles in as-cast condition is large enough for the heterogeneous nucleation of primary Si and/or eutectic Si, as shown in Fig. 1c,d. However, the size of AlP particles in melt spun conditions may still not be large enough for the heterogeneous nucleation of primary Si and/or eutectic Si, as shown in Figure S3 (Supplementary Information). The smaller size of primary Si and/or eutectic Si is mainly attributed to the higher cooling rates in melt spun conditions.

The formation of AlP particles can also be categorised in two cases: (i) prior to the eutectic reaction (eutectic Al and eutectic Si), the AlP particle forms from the liquid state, which can act as a heterogeneous nucleation site for primary Si. (ii) During the eutectic reaction (eutectic Al and eutectic Si), the AlP particle forms at the surface of eutectic Al, and thereby provides favourable conditions for the heterogeneous nucleation of eutectic Si on eutectic Al. In the present investigation, as shown in Figs 1 and 2, a large AlP particle was observed in the centre of primary Si (Figs 1c,d and 2a), indicating that this AlP particle most likely formed from the liquid state and is responsible for the heterogeneous nucleation of primary Si. However, as shown in Figs 3, 4, 5, 6, small AlP particles are also found at the interface between the Al matrix and eutectic Si: their geometry and location suggest that they are initially nucleated on the surface of the Al matrix. Furthermore, in this case, the perfect cubic to cubic orientation relationship between the observed AlP particle and eutectic Si, as shown in Fig. 6b, confirms that the AlP particle formed at the surface of Al matrix, and subsequently acted as an efficient heterogeneous nucleation site for eutectic Si. It has been reported that even for extremely low P concentrations and large undercooling the direct nucleation of Si on Al does not occur and that it is instead triggered by the formation of AlP under clean conditions^25^,^26^,^27^. It is the formation of AlP particles on the surface of the Al matrix that enhances the heterogeneous nucleation of eutectic Si, which is similar to the well-known fact that the adsorption of Al_3_Ti on the TiB_2_ particle promotes the heterogeneous nucleation of Al grain on the TiB_2_ particle and thereby enhances the grain refinement potency of the TiB_2_ particle^29^,^30^. The present investigation thus provides some novel insights into the heterogeneous nucleation of primary Si and/or eutectic Si in hypereutectic Al-Si alloys and can be used to further develop a heterogeneous nucleation mechanism based on adsorption.

## Methods

Al-18 wt.% Si alloys (wt.%, is used throughout the paper unless otherwise noted) without and with the addition of 0.03P were prepared using high purity (HP) Al (5N, 99.998), HP Si chips (6N), and an Al-3P master alloy. The chemical concentration of Al and Si was determined by an inductively coupled plasma atomic emission spectrum (ICP-AES) apparatus. The P concentration was determined with a Finnigan ELEMENT GD glow-discharge mass-spectrometer (GD-MS). The measured composition is listed in Table 1.

The Al-18Si alloys without and with the addition of 0.03P were melted in an electric resistance furnace and the melting temperature was kept at about 850 °C to ensure the incorporation of P at high concentrations. No degassing was performed prior to casting into a die mould.

Due to the fact that P is very likely to react with O_2_ (oxidation) and/or water during sample preparation, the samples for EBSD investigation were cut off by using a diamond saw without any cooling medium (e.g. water), and subsequently polished by using standard ion milling at 4 kV for 4 h. The EBSD investigation was performed using a Zeiss 1525 scanning electron microscope equipped with an EDAX EBSD system. The evaluation of scans was carried out using orientation imaging microscopy (OIM) software. The EBSD investigation can be used to determine the orientation relationship between the primary Si particles and the AlP particle.

The samples for TEM investigation were prepared using the standard lift-out technique in an Auriga CrossBeam Workstation (Carl Zeiss SMT)^31^. The device combines a field emission cathode with an EDX system (EDAX, SDD Apollo 40) and a high resolution focused ion beam (FIB) (Orsay Physics Cobra Ga^+^ ion FIB) in one instrument. The FIB acts as a nano-scale scalpel to produce well-defined cuts within the material. Electron and FIB-columns are arranged at an angle of 54°, which permits a direct observation on the processing within the SEM. Slicing was performed perpendicular to the sample surface. The direction of the cutting motion proceeds from the bottom to the top. After thinning the TEM samples, a low energy milling at a voltage of 2 kV was performed to minimize possible damage induced by Ga^+^ ions and re-deposition of material on the surface of the lamella.

HAADF STEM imaging and EELS were performed using a Nion UltraSTEM100 aberration corrected dedicated STEM. The microscope was operated at an acceleration voltage of 100 kV and an electron probe convergence semi-angle of 31 mrad, which resulted in an estimated minimum electron probe size of 0.8 Å. The cold field emission gun of the microscope has a native energy spread of 0.35 eV. The HAADF detector semi-angles were 83–185 mrad and the spectrometer collection semi-angle was 36 mrad. EELS maps where then created by integrating the EELS signal of each edge: Al *K* (1560 eV), Si *K* (1839 eV) and P *K* (2146 eV), over a suitable energy window after subtracting the preceding exponential background fitted with a power law. All EELS edges were identified following reference^32^. The intensities of the EELS maps displayed in Figs 3, 4, 5, 6 were displayed on a false colour scale, so that within each map, a low intensity (black) corresponds to a lower relative concentration, while increased contrast (colour) corresponds to an increase in elemental concentration.

## Additional Information

**How to cite this article**: Li, J. *et al.* Revealing heterogeneous nucleation of primary Si and eutectic Si by AlP in hypereutectic Al-Si alloys. *Sci. Rep.* **6**, 25244; doi: 10.1038/srep25244 (2016).

## Supplementary Material

Supplementary Information

## Figures and Tables

**Table 1:** The measured compositions of Al-18Si based alloy without and with 0.05P addition.

Si	P (ppm)	Al
18.00	0.44	Balance
18.00	300	Balance

(wt.%).
