# Supplementary material for: Revealing heterogeneous nucleation of primary Si and eutectic Si by AlP in hypereutectic Al-Si alloys
Source: Sci Rep. 2016 Apr 28;6:25244. doi: 10.1038/srep25244 (PMC4848514; doi:10.1038/srep25244)
Supplement: Supplementary Information [file srep25244-s1.doc]

# Revealing heterogeneous nucleation of primary Si and eutectic Si by AlP in hypereutectic Al-Si alloys

Jiehua Li^1^*, Fredrik S. Hage^2^, Xiangfa Liu^3^, Quentin Ramasse^2^, Peter Schumacher^1,4^

^1^ Institute of Casting Research, Montanuniversität Leoben, Leoben, A-8700, Austria

^2^ SuperSTEM Laboratory, SciTech Daresbury Campus, Keckwick Lane, Daresbury, WA4 4AD, UK.

^3^ Key Laboratory for Liquid-Solid Structural Evolution and Processing of Materials, School of Materials Science and Engineering, Shandong University, Jinan 250061, China.

^4^ Austrian Foundry Research Institute, Leoben, A-8700, Austria

*Corresponding authors:

J.H. Li: Institute of Casting Research, Montanuniversität Leoben, A-8700, Leoben, Austria.

Tel.: +43-3842-402-3304; Fax: +43-3842-402-3302.

# Email address: [**jie-hua.li@hotmail.com**](mailto:jie-hua.li@hotmail.com)

**Supplementary Information**


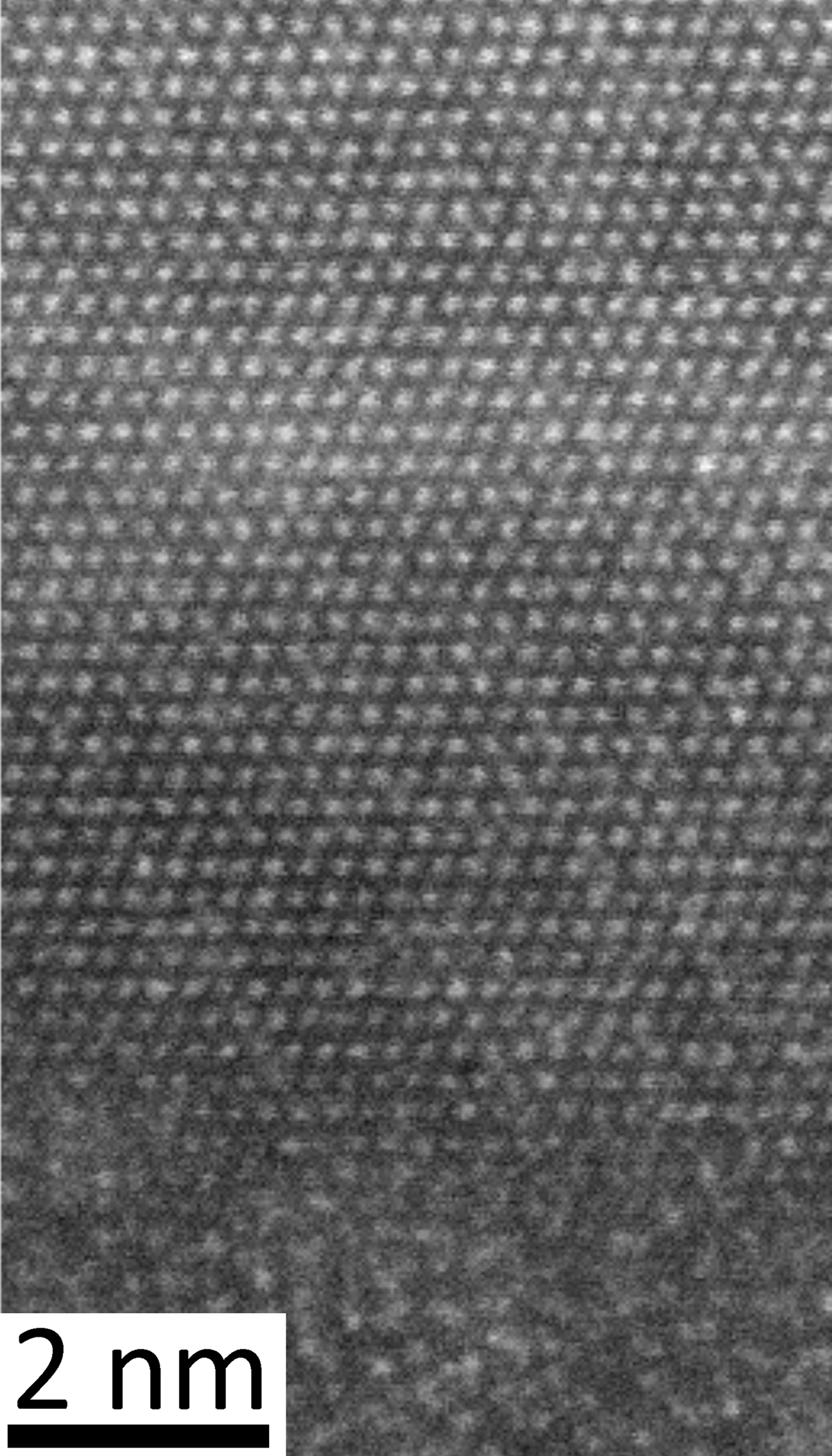


**Figure S1: High resolution HAADF STEM image of Al matrix in Al-18Si-0.03P alloy.** The Al matrix was tilted to the <011>_Al_ zone axis. This image is taken from the interface overlapping with the AlP particle (the no-lattice contrast at the bottom of the image is Si).

**
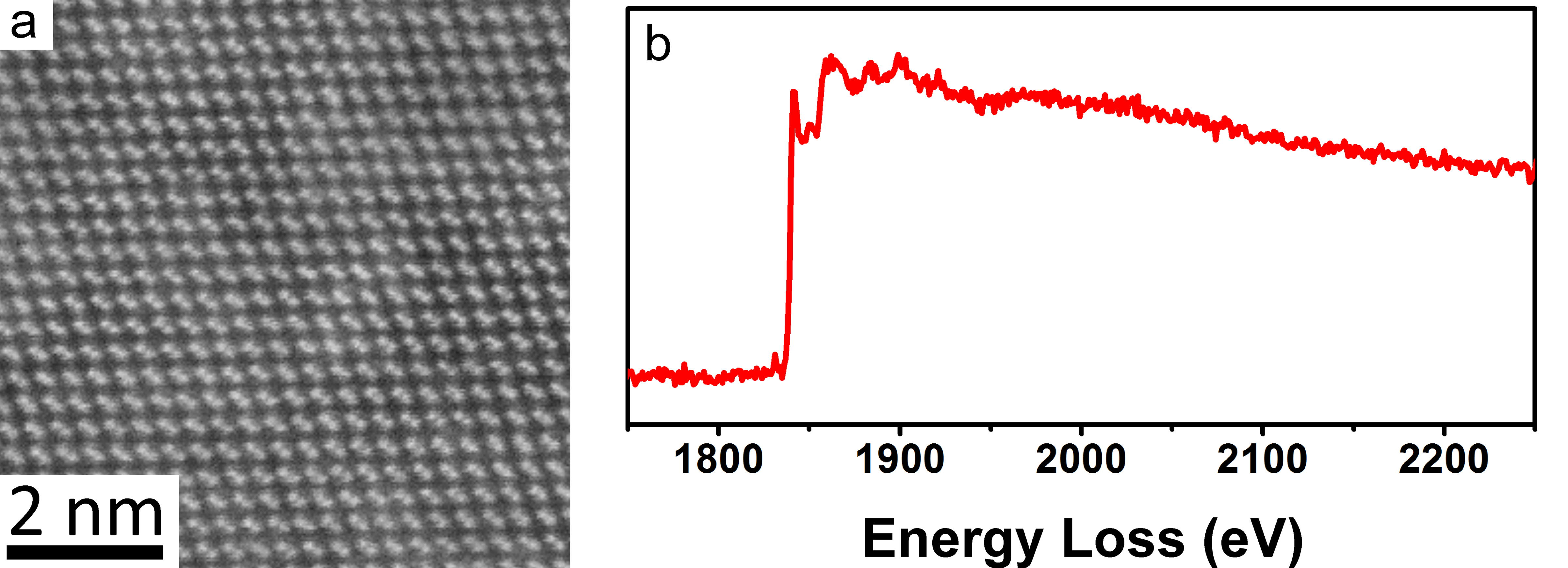
**

**Figure S2: High resolution HAADF STEM image (a) and EELS (b) of eutectic Si in Al-18Si-0.03P alloy.** The Si particle was tilted to the <011>_Si_ zone axis. No significant signal from P was detected within the Si particle.

For the melt spinning experiments, alloy charges of ~2.5 to 3 g were remelted in a quartz crucible (1 mm Ø orifice) and ejected (super heat ΔT = 125 K, ΔP = 100 mbar) under reduced He (200 mbar) atmosphere onto a Cu wheel rotating with a wheel speed of 15 m s^-1^, resulting in ribbons ~3 mm wide and ~80 µm thick. The cooling rate was estimated to be in the order of 10 6 K s^-1^.

TEM and high resolution TEM was performed using an image-side Cs-corrected JEOL-2100F operated at 200 kV.

Figure S3 shows SEM images of a melt spun Al-18Si-0.03P alloy. Figure sS3a and S3b show across section of the sample before FIB cutting. Figures S3c and S3d show across section after FIB cutting. The primary Si in melt spun condition is much smaller than that in as-cast conditions. However, no significant presence of AlP particles was observed in melt spun conditions, indicating that the higher cooling rate in melt spun condition (10 ^6^ K / s) increases the solubility of P in Al. Furthermore, in melt spun conditions, significant Si twins were also observed, as shown in Figure S4, which is in good agreement with what is observed within eutectic Si, indicating that the impurity-induced twinning (IIT) mechanism and / or the twin plane re-entrant edge (TPRE) mechanism are also active in the case of primary Si.

**
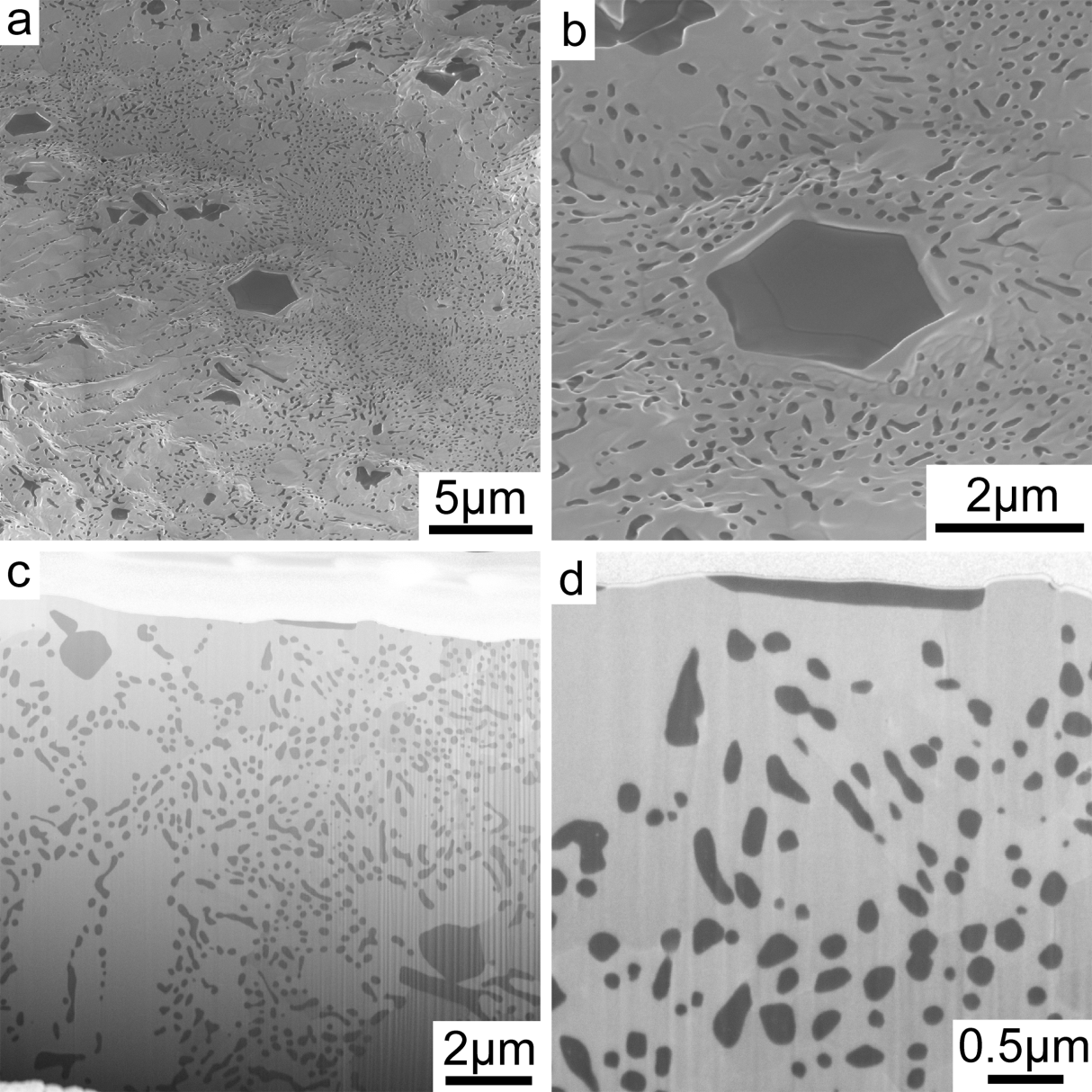
**

**Figure S3: SEM images of melt spun Al-18Si-0.03P alloy.** (a) (b) across section before FIB cutting, (c) (c) across section after FIB cutting. No significant AlP particle was observed in melt spun conditions. The primary Si in melt spun conditions is much smaller than that in as-cast conditions due to the higher cooling rate (10 ^6^ K / s).

**
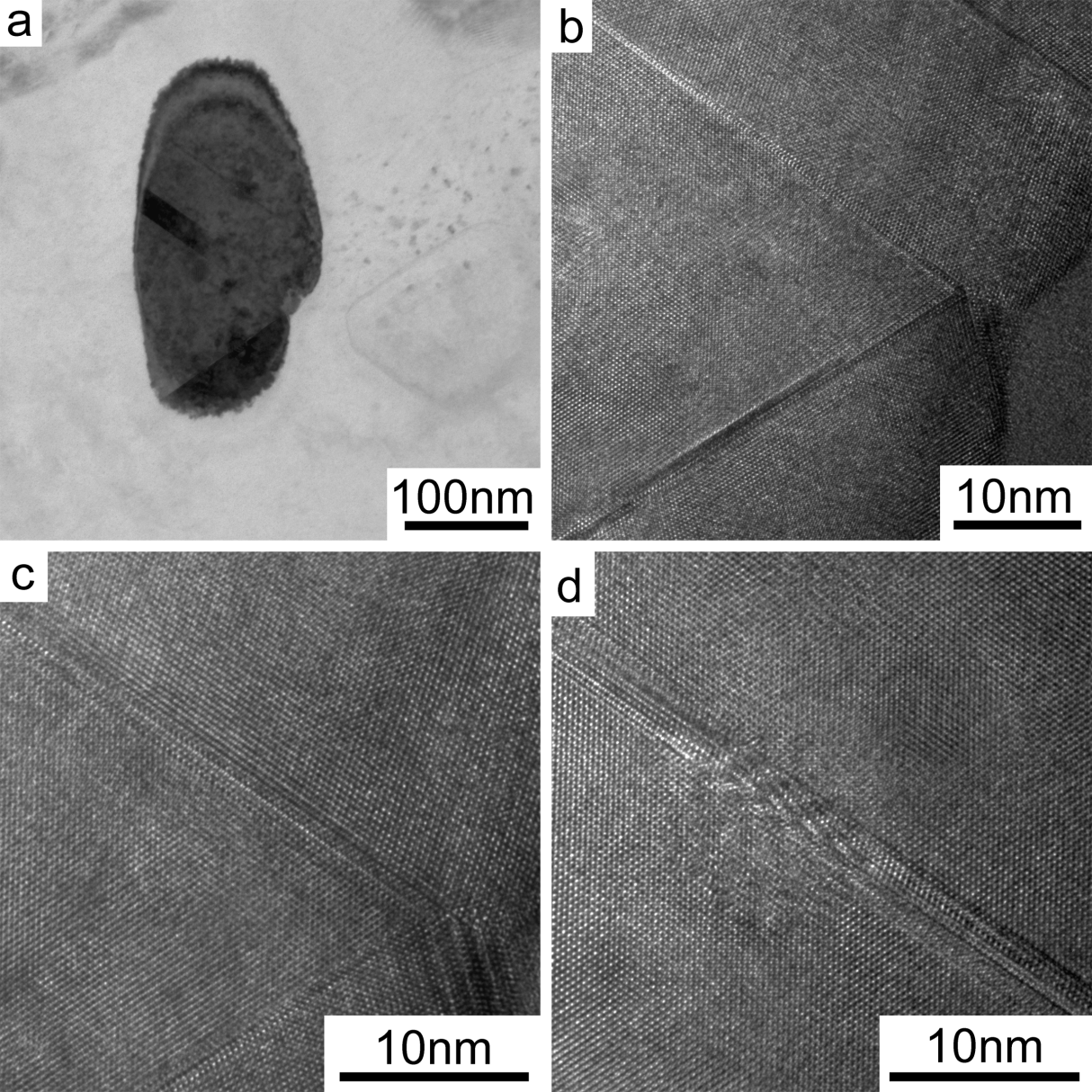
**

**Figure S4: TEM images of melt spun Al-18Si-0.03P alloy.** (a) shows the primary Si in melt spun conditions is relatively smaller than that in as-cast conditions. (b) (c) (d).show the Si twins within primary Si, which is in good agreement with that within eutectic Si, indicating that the impurity-induced twinning (IIT) mechanism and the twin plane re-entrant edge (TPRE) mechanism are also active in the case of primary Si.
